# A novel 4-arm DNA/RNA Nanoconstruct triggering Rapid Apoptosis of Triple Negative Breast Cancer Cells within 24 hours

**DOI:** 10.1038/s41598-017-00912-3

**Published:** 2017-04-11

**Authors:** Joline Tung, Lih Shin Tew, Yuan-Man Hsu, Yit Lung Khung

**Affiliations:** 1 Institute of New Drug Development, China Medical University, Taichung, Taiwan; 2 Regenerative Medicine Cluster, Advanced Medical and Dental Institute (AMDI) Universiti Sains Malaysia, Penang, Malaysia; 3 Department of Biotechnology, China Medical University, Taichung, Taiwan

## Abstract

Measuring at ~30 nm, a fully customizable holliday junction DNA nanoconstruct, was designed to simultaneously carry three unmodified SiRNA strands for apoptosis gene knockout in cancer cells without any assistance from commercial transfection kits. In brief, a holliday junction structure was intelligently designed to present one arm with a cell targeting aptamer (AS1411) while the remaining three arms to carry different SiRNA strands by means of DNA/RNA duplex for inducing apoptosis in cancer cells. By carrying the three SiRNA strands (AKT, MDM2 and Survivin) into triple negative breast MDA-MB-231 cancer cells, cell number had reduced by up to ~82% within 24 hours solely from one single administration of 32 picomoles. In the immunoblotting studies, up-elevation of phosphorylated p53 was observed for more than 8 hours while the three genes of interest were suppressed by nearly half by the 4-hour mark upon administration. Furthermore, we were able to demonstrate high cell selectivity of the nanoconstruct and did not exhibit usual morphological stress induced from liposomal-based transfection agents. To the best of the authors’ knowledge, this system represents the first of its kind in current literature utilizing a short and highly customizable holliday DNA junction to carry SiRNA for apoptosis studies.

## Introduction

Some of the quintessential characteristics of any customizable therapeutics should be non-immunogenic, low toxicity as well as high tissue specificity^1–4^ but attaining these attributes remains challenging at this stage. This is largely due to the wide choices available in innovative molecular/drug designs, ranging from nanoparticles^5, 6^ to drug-antibody conjugates^4, 7^. Yet not a single system can be truly beyond reproach^8, 9^. Of the many novelties advocated in literature, delivering RNA interference (RNAi) remains a strong contender for treating diseases at a cellular level by means of repressing and shutting down disease-causing genetic anomalies^10–13^. While RNAi technology currently represents the forefront in gene suppression from an academic standpoint, it does suffer from some drawbacks as well. High degradation rate within the cytoplasmic environment^14^ as well as the requirement for high dosage^15, 16^ had drawn much criticism from the scientific community. Chemically modified RNA may improve the circulation longevity^15^ but is highly susceptible towards immunogenic responses^17, 18^. Hence, the plasmid derivatives in the form of ShRNA (short hairpin RNA) are often promoted in its place for the maintenance of interfering RNA levels^19^. Nevertheless, the main problem remains in the selection of the most appropriate delivery mechanism into the cell. The most common mode of deliverance is via liposomal-based technology but the issues of cytotoxicity^20, 21^ as well as the lack in cell-specificity were serious enough to impede its development as a viable clinical option. While handling issues pertaining to cytotoxicity remains a tricky and daunting task, gaining high cell specificity is comparatively more straightforward. Many groups in the past had tried to covalently conjugate antibodies directly to liposomes^22–24^ but the problems of immunogenic responses towards the antibodies had plagued these hybrid systems right from the start^25, 26^. This had subsequently regressed antibodies-liposome hybrids (coined “immunoliposomes”) to the role of useful *in-vitro* tools. In contrast, DNA aptamers, with its lower level of immunogenicity^27^ as well as being more economically viable compared to antibodies, are often proposed as another alternative for cell targeting.

DNA aptamer are short strands of DNA that can readily self-hybridized with itself to present important tertiary structures. They can serve to bind to cell surface receptors and ultimately gaining entry into cell targets ﻿with high specificity﻿^28–31^. Indeed, synergizing both aptamer and RNAi had already gained much footing in literature and reports had already shown considerable success in recent years^10, 32–34^. Advantages of using aptamers over antibodies are that they are usually inexpensive and have a higher shelf life compared to antibodies^35^. They can be easily tailor-made through SELEX enrichment procedures and are more thermally stable. Compared to liposomal-based delivery^21, 36^, aptamers do not require any additional preparation steps other than purification prior to administration to cells and they do not typically induce any of the cytotoxicity compared to liposomal delivery^37^.

Much of the current research has reported the use of aptamers to deliver single antisense RNA (double stranded) and most often involved covalent conjugation of the ends of the DNA aptamer directly to the functional end of the RNA or as “chimeras”^10, 38^. These bioconjugated DNA/RNA nanocomplexes were then administered directly to the designated cell, and gene suppression was subsequently measured. However, the process of bioconjugation can be technically difficult in untrained hands, and RNA chimeras may suffer from potential immunogenic responses. Coupled by the fact that many of these systems only dealt with a single gene target, this may not be as efficient compared to multiple gene targets being singled out simultaneously. It was with these thoughts that we proposed this DNA/RNA nanoconstruct that was designed to simultaneously carry multiple copies of antisense RNA strands into cells. Borrowing from the concepts of the self-assembled holliday junction as demonstrated by Li *et al*. delivering fluorophores^39^ as well as complex DNA/RNA machinery by Lee *et al*.^40^, w﻿e assembled a four-armed nanoconstruct in a single pot fashion and an aptamer (AS1411) was selected to anneal towards one of the arms via a sequence specific ‘sticky’ end. The remaining three arms comprised of overhanging sequences that were complementary to its individual antisense RNA sequences and the respective RNA strands were then hybridized to these selective regions via DNA/RNA hybrid duplex.

**Figure 1:**
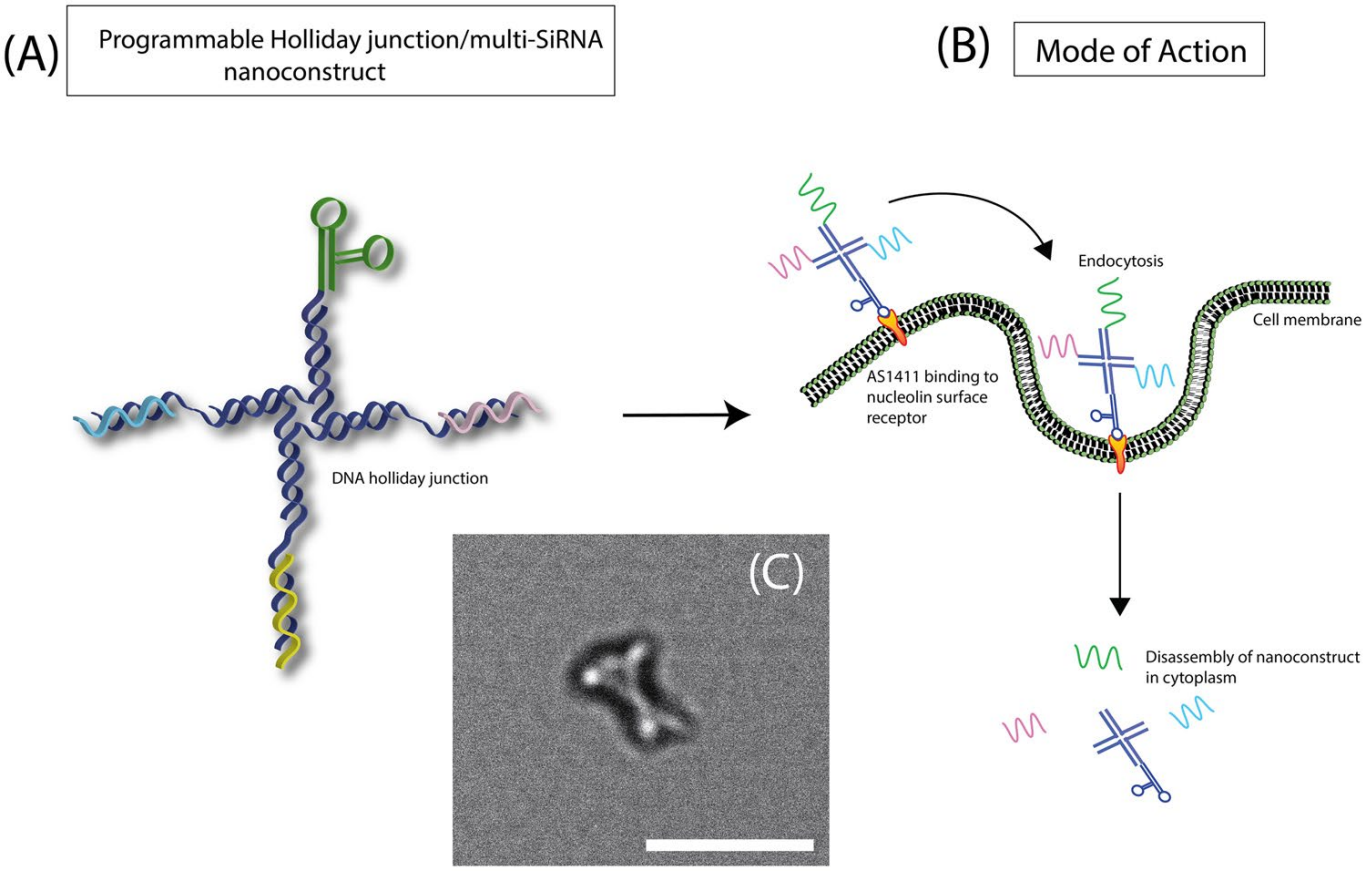
(**A**) Graphical illustration of the holliday junction DNA nanoconstruct carrying multiple antisense RNA. (**B**) Mode of transfection by which the nanoconstruct gain entry and subsequent disassembly within the cell and (**C**) inset with white arrows showing the transmission electron microgram exhibiting the cross-like features upon full assembly (white bar represents 50 nm). See Supplementary Figure 1 for the sequence of the nanoconstruct.

In short, all the strands were introduced into a single PCR tube and placed in a thermocycler and the temperature slowly reduced from 95 °C to 4 °C in order to self-assemble into its final holliday configuration as shown in Fig. 1A. This process of rapidly assembling took no more than an hour. Herein, we were able to show that this nanoconstruct was able to induce high cancer cell apoptosis within 24 hours and could be tailored to suppress any gene of interest by the mere change of the sequence selection without relying on overly complicated bioconjugation processes.

## Results and Discussion

Four strands of DNA were specially designed to host three separate antisense RNA strands while leaving a single arm for hybridization to an aptamer DNA (see Fig. 1A). We had especially devised the nanoconstruct to be a DNA/RNA duplex system due to several considerations but principally for its lower immunogenicity compared to RNA/RNA as reported by Afonin *et al*.^41^. One other important consideration was the potential cleavage by cellular Ribonuclease H1 of the DNA/RNA duplex within the cell cytoplasm. To mediate this, the system was designed in such a way that the 5′ end for all three RNA strands (19 bases) was approximated less than 5 nm relative to the center of holliday junction. Molecular dynamics simulation on the cellular ribonuclease H1 interaction with DNA/RNA hybrid strand revealed that the binding process would require a surface area of 177 nm^2^ on site^42^. But it is essential that the DNA region to be free of any structured loops (such as hairpin loops etc) in order to accommodate the binding domain of the ribonuclease^43^, which would not be in our case due to the immediate flanking of the RNA’s 5′ end with DNA. The key recognition of the binding domain for Ribonuclease H1 to the width of the minor groove had been reported to be around 7.5 Å^44^ and is highly intolerant towards other dimensions. The holliday junction DNA right next to the 5′ RNA/DNA duplex antisense strand would distort and rigidize the backbone of the DNA/RNA which could offer resistance towards ribonuclease H1 cleavage. Moreover it was already well-established that structured RNA tends to cleave off significantly slower than unstructured RNA^45^. Hence, we postulated that the cross-like contorted nano-architecture could offer much steric hindrance towards the ribonuclease antisense and this would also help to reduce the overall processivity for the ribonuclease antisense to work on cleaving the DNA/RNA. It is important to note that if cleavage had indeed occurred for the DNA/RNA duplex, calculations from closest neighbor parameter would shown that the fragmented RNA oligomers were incapable of forming stable duplex with mRNA at 37 °C. It was therefore with these considerations that we proceeded with unmodified RNA strands rather than chemically modified species in order to reduce toxicity and facilitate for rapid clearance from the cellular system. The successful attainment of protein repression in later section of this manuscript had helped to reinforce these notions.

For this work, we had chosen AS1411 an aptamer that was widely reported in literature that would bind to the nucleolin protein on the cell membrane^46, 47^. Nucleolin was highly expressed in cancer cells and they were often localized on the outer-membrane while their surface presentation was not typically found on non-cancer targets^48^. The rationale was that upon the complete assembly of the nanoconstruct, the aptamer would bind to the cell surface to facilitate for uptake (see Fig. 1B) and once within the cellular environment, the nanoconstruct would undergo disassembly and disseminate its antisense RNA packages. Three protein targets, Akt1, MDM2 and Survivin, were selected as candidates for suppression as they were all responsible for maintaining the longevity of cancer cells. Both expressions of AKT1 and MDM2 proteins, following along a single pathway, suppress and ubiquintinate the important p53 protein which subsequently arrested apoptosis in cancer cells^49^. Hence suppressing them may in turn trigger the events of apoptosis. In conjunction to this, Survivin is an apoptosis inhibitor by means of inactivating caspase protein family and the liberation of caspase would in turn promote apoptosis^50^. It was with these thoughts that simultaneous repression of these three protein targets may aid in inducing rapid cell apoptosis.

**Figure 2:**
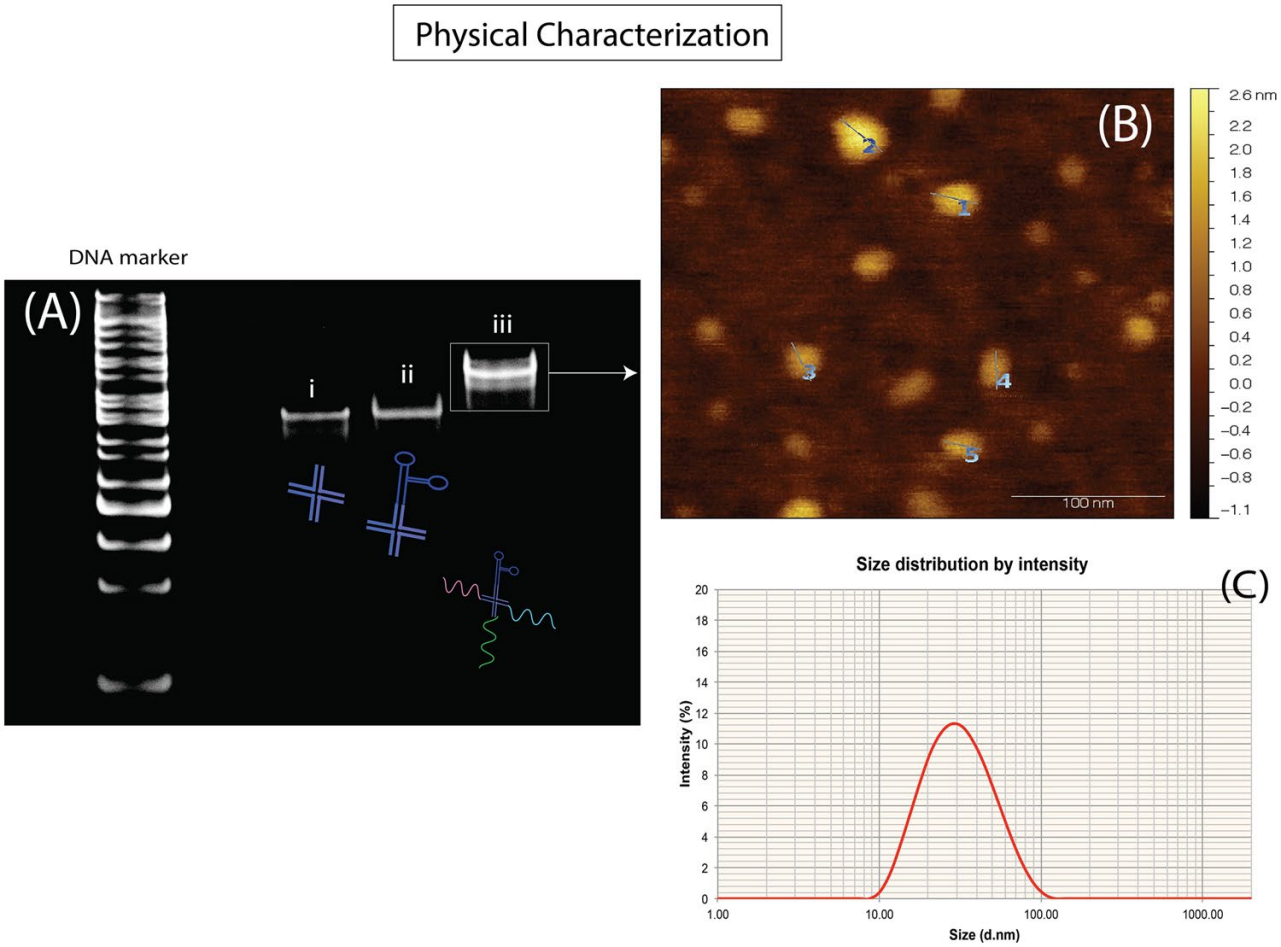
(**A**) Acrylamide gel electrophoresis and staining of the nanoconstruct (i) the four arms holliday junction strands without the aptamer, (ii) nanoconstruct with the aptamer strand and (iii) nanoconstruct with aptamer as well as all three antisense RNA hybridized to the overhanging sequences. Atomic force microscopy (**B**) and dynamic light scattering (**C**) had revealed that the nanoconstruct was approximately 28.64 ± 2.53 nm.

In a one step process, four strands of the holliday junction DNA strands, one strand of the AS1411 aptamer and three strands of the antisense RNA (Akt1, MDM2 and survivin) were mixed in equimolar concentration within a single PCR tube and were subjected to a melting temperature of 95 °C before allowing all oligomers to reassemble via complementary pairing with the slow gradual reduction of temperature to 4 °C. In order to validate the proper assembly of the full nanoconstruct, non-denaturing acrylamide gel electrophoresis was also performed (as shown in Fig. 2A) where the various strands were discerned under a UV-Transilluminator.

As observable in Fig. 2A, the nanoconstruct with only the four holliday junction arms (Fig. 2A(i)) migrated the fastest while there was a slight retardation (8% polyacrylamide gel) in migration speed when the aptamer was introduced (Fig. 2A(ii)). What was notable was that upon the addition of the three antisense RNA strands, there was a increase in size that may be explained by the successful hybridization of the antisense RNA to the DNA holliday junction as predicted. The DNA ladder cannot be taken as a correct size indicator due to the fact that branched DNA are motionally impeded in the gel matrix^51, 52^ while the retardation of the entire DNA/RNA hybrid nanoconstruct was expected as RNA tends to move slower in gel electrophoresis compared to DNA species^53^. The regions within the non-denaturing gel was then excised and purified via spin-column and atomic force microscopy (AFM) was subsequently performed to verify the sizing of the nanoconstruct. As shown in Fig. 2B, the nanoconstruct was determined to be at approximately 28.64 ± 2.53 nm while dynamic light scattering studies (Fig. 2C) had shown that the hydrodynamic size of the nanoconstruct approximated close to 31 nm. Transmission electron microscopy was also performed on these nanoconstructs and the observations were in tandem with the findings above (Fig. 1C). Furthermore, the experimental sizing of this nanoconstruct was in full agreement with the hypothetical approximations based on calculations of closest neighbor parameter as performed earlier.

**Figure 3:**
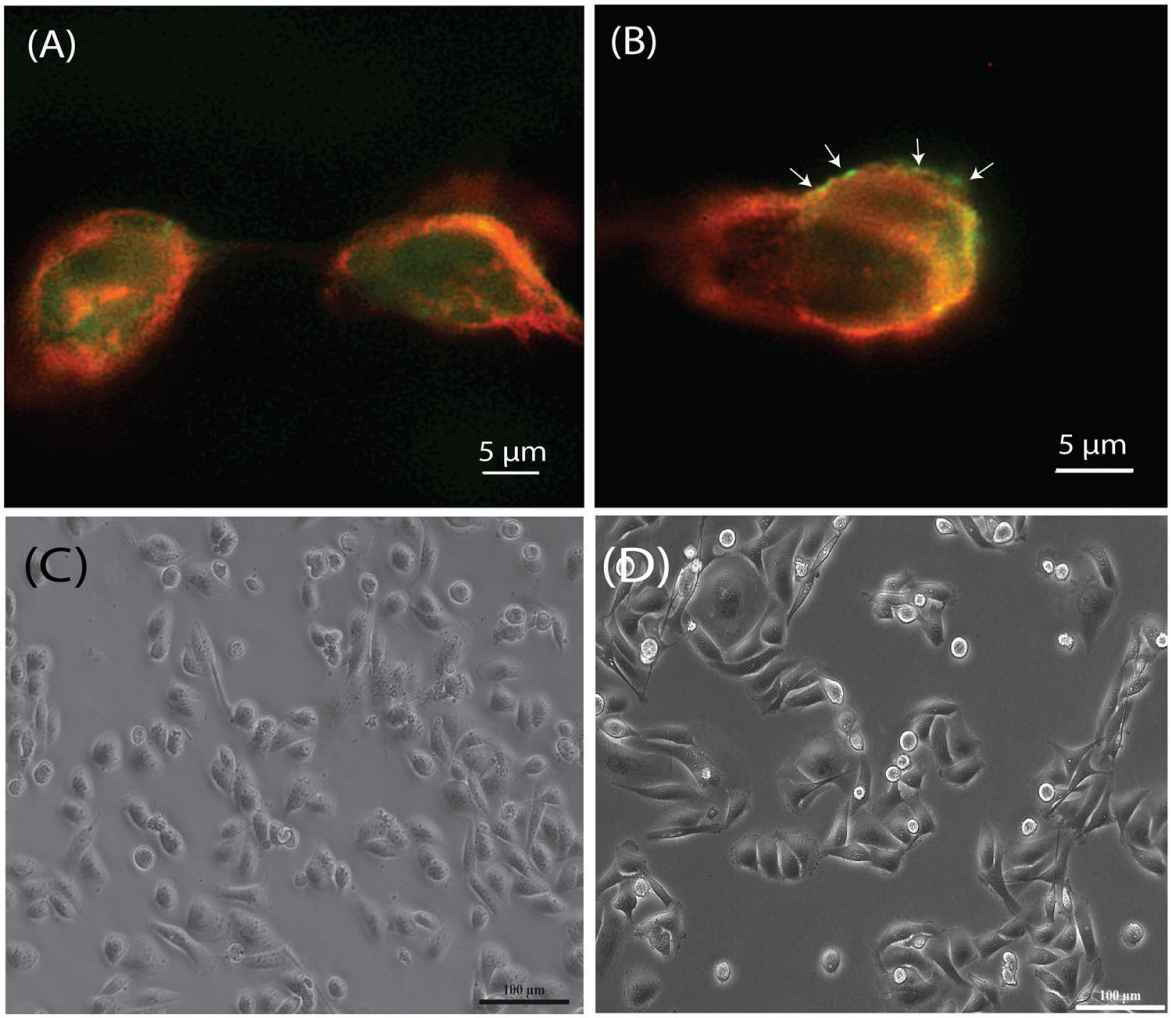
Stimulated emission depletion (STED) microscopy on MDA-MB 231 breast cancer cells incubated with FITC tagged nanoconstruct after 2 hours. (**A**) FITC tagged nanoconstruct with aptamer gaining entry into cells hence resulting in fluorescence within the cytoplasm while (**B**) FITC tagged nanoconstruct without the aptamer was unable to transfect the cells and fluorescence was found to be localized near the outer membrane. Light microscopy of the 231 cells at the 4-hour mark after transfection with (**C**) lipofectamine with the three single strands antisense RNA and (**D**) the nanoconstruct with AS1411 aptamer and three hybridized antisense RNA. Note the morphological disturbance induced by the presence of the lipofectamine where overall became stouter with lesser protrusions radiating out.

Using a holliday junction decorated with the AS1411 aptamer, a FITC tagged DNA sequence homologous to that of the antisense RNA belonging to the MDM2 was used to hybridized to the nanoconstruct and MDA-MB 231 cells were incubated for 2 hours. This was to examine if the cells were able to uptake nanoconstruct on the virtues of the AS1411 aptamer as selected. As shown in Fig. 3A, stimulated emission depletion (STED) microscopy performed on the FITC tagged nanoconstruct carrying the AS1411 aptamer was able to gain entry into the cytoplasmic region of the cell while a FTIC-tagged nanoconstruct without the aptamer was found to be localized at the outer peripheral regions of the membrane (as shown in Fig. 3B, white arrows). Hence the results confirmed that these nanoconstructs were indeed able to gain entry into the cells with the AS1411 aptamers. In conjunction to this, we had also decided to compare the morphological outlook of administrating our nanoconstruct to cells and how cellular morphology may differ when using lipofectamine. As shown in Fig. 3C, the three antisense RNA strands were prepared with lipofectamine at a final concentration of 16 picomoles concentration and incubated for 4 hours while Fig. 3D shows the 231 cells incubated with the nanoconstruct at 16 picomoles as well and incubated at 4 hours. The light microscopy images revealed that 231 cells after incubation with lipofectamine had shown much morphological duress as the cell shape was much stouter compared to those from the nanoconstruct. On the other hand, the transfection event from the nanoconstruct was visually observed to have very little effect of the overall morphological of the 231 cells after 4 hours and this was taken to have had induced less stress to the cells.

**Figure 4:**
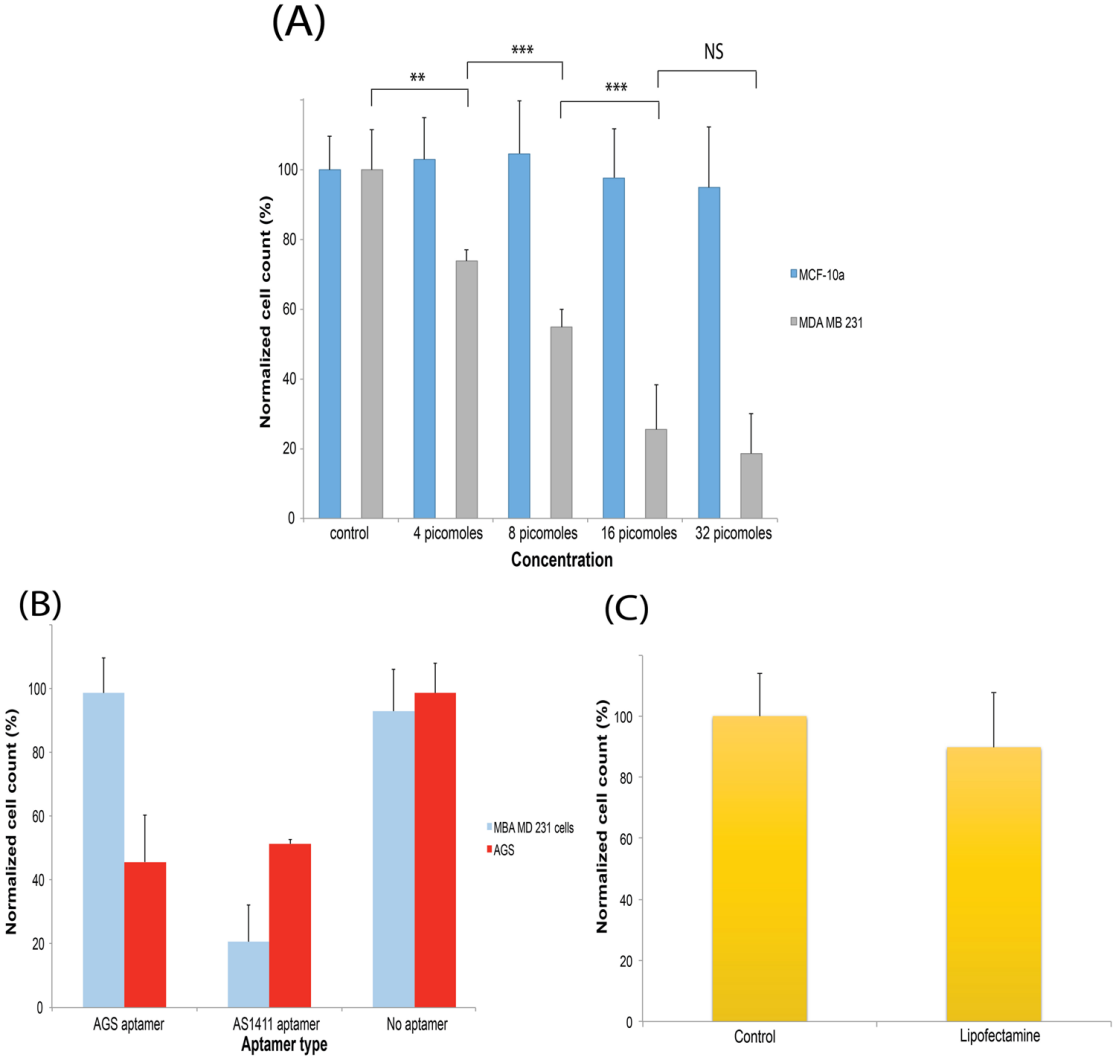
Apoptosis level on (**A**) MDA-MB 231 cells after 24 hours showing that at 32 pmoles, the nanoconstruct was able to induce cellular apoptosis of up to 82%. (**B**) 24 hour cell count from the incubation of the MDA-MB 231 and AGS cells with nanoconstruct that was constructed with the AGS-selective aptamer, AS1411 and when no aptamer was used. The absence of aptamer did not shown any appreciable reduction in numbers for both cells types. (**C**) 16 picomoles of single stranded antisense RNA was packaged with commercial lipofectamine 3000 and 231 cells were transfected without any notable changes in cell numbers after 24 hours.

In a series of dose dependent studies, the full nanoconstruct carrying the three apoptosis inducing antisense RNA was purified from non-denaturing polyacrylamide gels and were added at 4, 8, 16 and 32 pmoles to MDA-MB 231 cells for a period of 24 hours. After the incubation period, cells were collected and the cell numbers were determined using the hemocytometer. Figure 4A shows the extent of the apoptosis level induced by the nanoconstruct from the dose dependent studies. At 4 pmoles and 8 pmoles, we were able to observe a reduction in cell number to 73.79% ± 3.23% and 54.82% ± 5.18% respectively. However at 16 pmoles and 32 pmoles, the drop in cell count was observed to be at 25.51% ± 12.86% and 18.62% ± 11.51% but both sets of observations were statistically insignificant relative to one another. This suggested that at 32 pmoles, the threshold of efficiency was met (no notable reduction was also observed for at 64 pmoles) and any further increase in concentration was deemed unnecessary.

In order to further demonstrate the stringent cell-type selectiveness of this nanoconstruct, two other cell types were incubated with the apoptosis-inducing nanoconstruct at the dosage of 16 pmoles. As shown in Fig. 4B, non-tumorigenic epithelial cell line MCF-10A cells were known to have little expression of nucleolin on its surface membrane and was therefore employed to act as a positive control. After a 24hr incubation with the nanoconstruct, the MCF-10A cells showed very little change in its overall cell number. On the other hand, the gastric cancer cell line AGS was also engaged in this study and after 24 hours, there was a marked reduction in cell number observed (48.52% ± 4.59%). This had shown that the nanoconstruct was indeed selective toward cancer cell types that had AS1411 expression on its outer membrane. However, when no aptamer was assembled into the nanoconstruct, both cell types did not exhibit any notable changes in cell number after incubation for 24 hours. Once again, this had suggested that the transfection was indeed mediated by the presence of the aptamer on the nanoconstruct.

Cell count for lipofectamine-based transfection was performed using the three single strand antisense RNA at 16 picomoles. As these antisense were unmodified, we did not envisage for any high efficiency from this delivery event and this was as shown in Fig. 4C whereby the changes to cell number was only marginal (88.78% ± 27.21%). This had confirmed that the arrangement of this DNA/RNA branch duplex can indeed drive apoptosis more efficiently compared to delivering naked unmodified antisense RNA.

**Figure 5:**
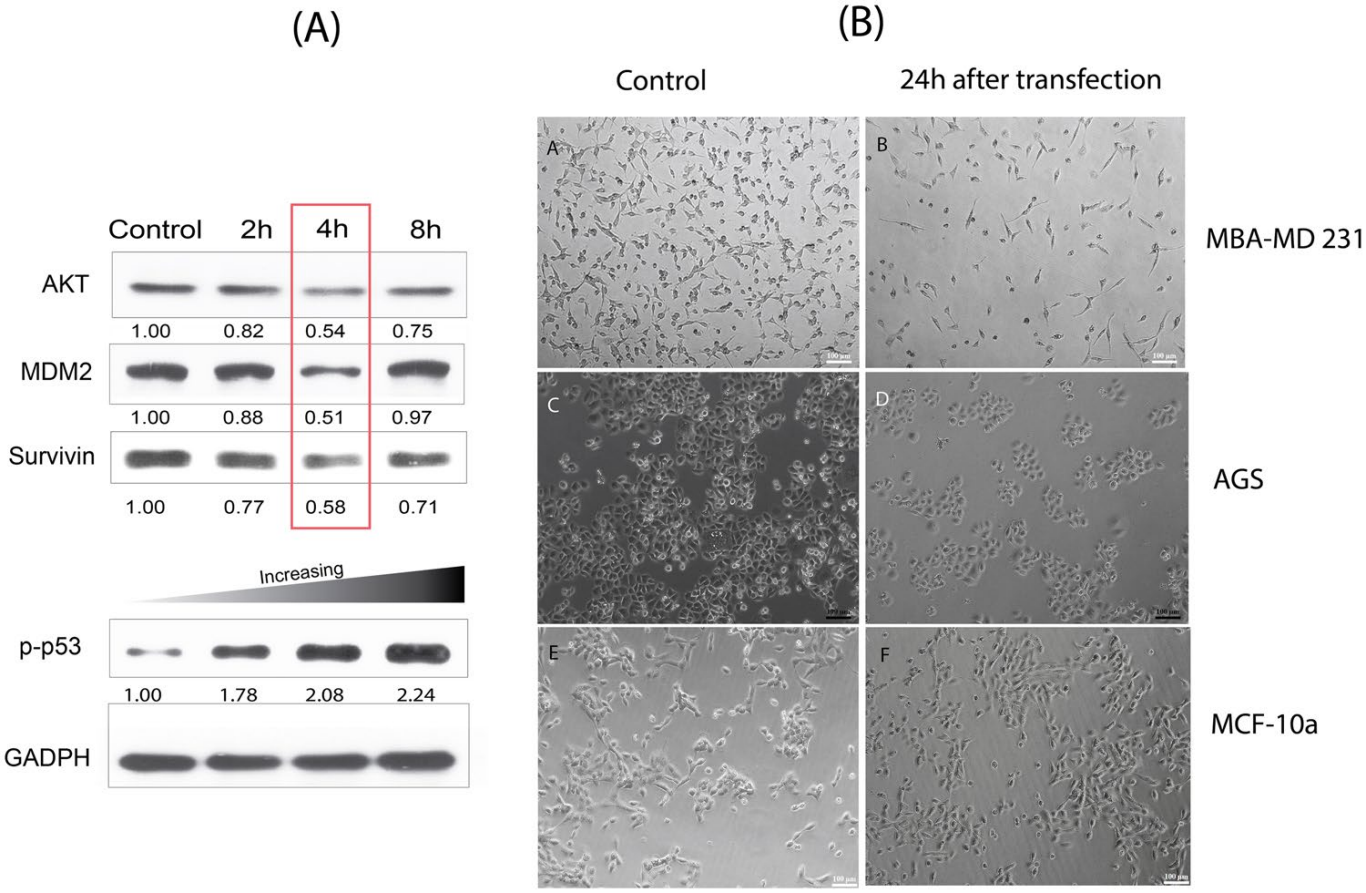
(**A**) Levels of suppression of Survivin and expression of p53 for 2 h, 4 h and 8 h for MBA-MD 231 cells. (**B**) Light microscopy images of the three cell types using nanoconstruct with the AS1411 aptamer. Cell population had been observed to reduce for the MBA-MD 231 cells and AGS while the non-tumorigenic cells MCF-10a did not exhibit any appreciable loss in cell number as well as cell morphology.

As mentioned three antisense RNA protein were selected under special consideration that all the genes contribute towards cell death. AKT and its downstream MDM2 are proteins that regulate the expression of p53 and the suppression of these genes would upregulate phosphorylated p53 expression levels that would contribute towards cell apoptosis. On the other hand, survivin was another protein that inhibits caspase activity, thus contributing to the overall unnatural longevity of cancer cells. Hence, by suppressing all these three genes, it was in principle possible to induce rapid cell death. In Fig. 5A, the suppression of these three proteins was characterized with immunoblotting for a course of 2 h, 4 h and 8 h to evaluate the time course of the repression. At the fourth hour mark, we noticed that all three proteins were suppressed to between 42–49%. Their collective reduction in level had demonstrated that the three antisense strands had been successfully delivered into the cell. The recovery of their expression level collectively after 8 hours was subsequently observed and this was within our expectations, as the antisense RNA strands used in this study did not have any special chemical modification and would undergo rapid degradation. In conjunction with the increasing up-regulation of phosphorylated p53 level over the 8 hour period, this had strongly implied that while the suppression was a relatively transient affair, the initial shock imposed by repressing these three proteins at the same time were sufficiently disruptive enough to trigger series of cell apoptosis events, ultimately contributing to cell death of up to 82% at the 24 hour mark as far as quantification was concerned. Our observations may defer from the important report by Kracikova *et al*.^54^ by which that the maintenance of high p53 levels was deemed as a prerequisite for inducing cell apoptosis. However, this study could not be directed comparatively with that report as we had deliberately suppress three proteins in a simultaneous fashion although it is important to note that this was not the first time that this simultaneous repression of multiple protein target was attempted^55, 56^. Nonetheless the general consensus was that multiple protein suppression in a concurrent fashion could indeed shortened the time-line required for apoptosis (24 hours)^56–58^ and our findings here were no different.

Furthermore, in order to demonstrate that this nanoconstruct was easily programmable as a proof of concept, we decided to introduce three separate RNA antisense strand (8 picomoles) that would simultaneously suppressed the same metastatic Twist protein and we were able to obtain an even early repression at the 2 hour mark of up to ~77% (see Supplementary Figures 2 and 3). Even the downstream vimentin protein was also repression by upstream suppression of the Twist protein for up to 8 hours (see Supplementary Figure 3).

## Conclusion

Herein, we had demonstrated the feasibility of using a small holliday junction-type nanoconstruct to simultaneously delivery three antisense RNA strands for the suppression of AKT, MDM2 and Survivin proteins *in-vitro.* While the detected suppression event was relatively transient, this package was sufficiently detrimental towards the contribution of rapid apoptosis within 24 hours. Antisense RNA selected for this work was unmodified with the intentions for facilitating their rapid clearance from the biological system. Preliminary studies had shown that the administration of the nanoconstruct and the uptake did not affect its morphological characteristics compared to conventional lipofectamine based technologies and the nanoconstruct was especially cell-specific by merely interchanging the aptamer sequence. By assembling the nanoconstruct in the form of four immobile arms of the holliday junction, we believed that the structural arrangement may help to negate the effects of Ribonuclease cleavage which in turn confer certain structural stability. Based on the results garnered in this paper, holliday junction type architecture may present itself as another alternative for designing antisense therapeutics and work on *in-vivo* models would follow shortly.

## Methods and Materials

Unless otherwise specified, all chemicals and reagents were used as received. Primary antibodies were purchased from the following sources: MDM2 (Catalog Number: GTX100654) was purchased from Genetex, Survivin (Catalog Number: NB500-201) was acquired from Novus Biologicals and AKT (Catalog Number: #4691) was obtained from Cell Signalling. Anti-rabbit IgG, HRP-linked Antibody (#7074) was purchased from Cell Signalling as well. DMEM/F12 cell culture media, EGF (20 ng/ml), Insulin (10 ug/ml) was purchased from Invitrogen while Hydrocortisone (0.5 mg/ml) was from Sigma. 5% Horse Serum was acquired from HyClone Donor Equine Serum (#SH3007403) while Fetal Bovine Serum was purchased from Gibco**®**.

### Cell culture

All cell cultures were incubated in 5% CO_2_ incubator at 37 °C. MDA-MB-231 cells were cultured in DMEM-F12 supplemented with 10% fetal bovine serum and 1% penicillin-streptomycin. 90% RMPI 1640 medium, 10% fetal bovine serum and 1% penicillin-streptomycin was used to maintained AGS cells. Upon reaching 80% confluency, the cells were treated with trypsin after washing with PBS (3 times). The detached cells were centrifuged at 1000 rpm for 5 minutes. The pellets were then resuspended with growth medium at appropriate dilution, and cultured in a new 75 ml polystyrene cell culture flask.

The medium recipe for MCF-10A cells was adapted from Brugge’s lab. In brief, MCF-10A cells were grown at 37 °C incubator with 5% CO_2_ with DMEM and Nutrient Mixture F12 (Ham) (Invitrogen) with high glucose containing L-glutamine, pyridoxine hydrochloride, and HEPES buffer supplemented with 5% Donor Equine Serum (Hyclone), 20 ng/ml Epidermal Growth Factor, 0.5 mg/ml Hydrocortisone, 10 ug/ml Insulin, and 1% penicillin-streptomycin.

### Nanoconstruct assembly and purification

All DNA and RNA were obtained from Invitrogen**™** unless otherwise specified. To assemble the holliday junction nanoconstruct, the following sequences were selected to form a holliday junction with 4 overhanging ends that are complementary of the the individual RNA antisense strands and the respective aptamer sequence are also as listed below:

Holliday Junction 1 = 5′TTTGTGCAGCCAACCCTCCGTGTGTGTGCCATAGTGCATTGCGAGAGAGAG 3′

Holliday Junction 2 = 5′ATACTATCAGATTTGTGGCTTTCCTTTGCATTCGGACTATGGCACACACAC 3′

Holliday Junction 3 = 5′GCAGTGGATGAAGCCAGCCTTAAGGCCCGTGCTCACCGAATGCAAAGGAAA 3′

Holliday Junction 4 = 5′GGGGGGGGGGGCTCTCTCTCGCAATGCTGAGCACGGGCCTTAA 3′

AS1411 (nucleolin) aptamer = 5′ GGTGGTGGTGGTTGTGGTGGTGGTGG CCCCCCCCCCC 3′^59^

AGS cell specific aptamer = 5′ CGACCCGGCACAAACCCAGAACCATATACACGATCATTAGTCTCCTGGGCCG CCCCCCCCCCC 3′^60^

Three strands of antisense RNA was selected from well-cited sources in literature and was shown below:

AKT: 5′ GGAGGGUUGGCUGCACAAA 3′^61, 62^

MDM2: 5′ GCCACAAAUCUGAUAGUAU 3′^63, 64^

Survivin: 5′ GGCUGGCUUCAUCCACUGC 3′^36, 65^

To assembly the full nanoconstruct, all received oligomers (DNA and RNA) were firstly resolubilized in Rnase Free DEPC water (Invitrogen**™**) and 1 nmol for each strands (seven in total) were collectively pooled to a PCR tube and annealing buffer (10 mM Tris, pH 7.5–8.0, 50 mM NaCl, 1 mM EDTA) was added to bring the mixture to a final volume of 100 μl. The oligomer mixture was then mixed well before subjecting to the thermocycler (Biorad MJ Mini-personal Thermal Cycler) to 95 °C and held for 10 minutes. The temperature was then slowly reduced to 4 °C over 30 minutes before purification.

The nanoconstruct was purified using an 8% native polyacrylamide gel in Tris/Borate/EDTA buffer. The gel was sectioned out according to the size of the fully assembled nanoconstruct (a lane was stained with EtBr as a guideline). The sections were crushed and transferred to Nanosep® MF centrifugal devices and spun at a 17949 × *g* for 10 mins. All purified nanoconstruct was quantified with UV-Vis Spectrophotometer (Eppendorf Biophotometer plus) at 260 and 280 nm. The “roadmap” of the nanoconstruct was as shown in Supplementary Figure 1.

### Cell count analysis

For all cell types, the cells were seeded at a density of 2 × 10^4^ in a 24-well plate and then transfected with the nanoconstruct at the nanoconstruct concentrations of 4, 8, 16 and 32 picomoles (plated out in quadruplicates) for 24 hours. The cells were washed three times with phosphate-buffered saline and resuspended with 0.05% Trypsin-EDTA for ~15 mins. After which, 10 μl of the cell suspension was loaded into a hemocytometer and counted according to the manufacturer’s protocol. Statistical analysis of differences was performed via a one-way *ANOVA* analysis whereby P value of ≤0.05 was taken as statistically significance.

### Immunoblot

For immunoblotting, the cells were seeded at a density of 1 × 10^6^ and co-incubated with the nanoconstruct. Total protein concentration was determined by bicinchoninic acid assay (Thermo Fisher). Whole cell lysates were resolved by SDS-PAGE and transferred onto polyvinylidene fluoride membrane (Milipore) using a wet transfer system (Hoefer) with a Tris-glycine buffer containing 20% methanol. The membrane was then blocked with 3% bovine albumin serum in Tris-buffered saline with 0.1% Tween-20 (TBST) for an hour room temperature and gently washed twice with TBST. The membrane was probed with antibodies against Akt (Cell Signaling), MDM2 (Genetex), phosphorlayted-p53 (Novus Biologicals), and GADPH (Genetex) for ~16–18 hours at 4 °C and washed three times for ~10 mins with TBST. Afterwards, the blot was detected with HRP-linked Anti-rabbit IgG antibodies (Cell Signaling) for an hour at room temperature and followed by three washes with TBST for ~10 mins. The blot was developed using Immobilon Western Chemiluminescent HRP Substrate (Milipore).

### Negative staining for transmission electron microscopy

The following staining protocol was adapted from Sir William Dunn School of Pathology. In brief, Uranyl acetate was dissolved in deionized water to prepare a 2% stain solution. The nanoconstruct was diluted by 10-folds in DEPC-treated water. Equal ratios of the stain solution and nanoconstruct were combined. The mixture loaded onto the grid for ~30 seconds and then absorbed with filter paper. Transmission electron microscopy was performed on Hitachi HT7700 platform at an acceleration voltage of 70 kV.

### Atomic Force Microscopy

Atomic force microscopy (AFM) images were acquired using Bruker Dimension Icon AFM system using an in-build AFM tapping mode. Scan area on the surfaces were of 0.3 μ m × 0.3 μ m and the scan speed was set at 0.5 hz with the integral and proportional gain set at automatic mode. Post image processing was performed with Gwyddion MacOS version 2.38.

### Dynamic Light Scattering

Particle size of the nanoconstruct was determined using a disposable plastic cuvette through the Zetasizer Nano (Malvern #ZS90) at room temperature using DEPC water as the solvent.

### Super Resolution Microscopy

To evaluate the uptake of the nanoconstruct, the four arm holliday junctions with the AS1411 aptamer was assembled without antisense RNA but was replaced with a FITC-tagged complementary strand to holliday junction 2 (5′ GCCACAAATCTGATAGTAT 3′). The fluorescence labeled nanoconstruct (16 picomoles) was administrated to MDA-MB-231 cells at density of 1 × 10^6^. After 2 hours, the cells were washed 3 times with PBS followed by the nominal fixation protocol with paraformaldehyde and stained with Alexa Fluor® 594 phalloidin before mounting on cover slips. The surfaces were then examined under Nikon® N-SIM super resolution microscopy using using an oil immersion objective lens CFI SR at laser wavelength of 488 nm and 561 nm for the visualization of the nanoconstruct carrying FITC and the cell membrane respectively. Image processing was done with NIS Elements Revolutionizes Imaging software (Nikon Corporation, Shinagawa-ku, Tokyo, Japan).

## Electronic supplementary material

Supplementary Information
