# Supplementary material for: A novel 4-arm DNA/RNA Nanoconstruct triggering Rapid Apoptosis of Triple Negative Breast Cancer Cells within 24 hours
Source: Sci Rep. 2017 Apr 11;7:793. doi: 10.1038/s41598-017-00912-3 (PMC5429792; doi:10.1038/s41598-017-00912-3)
Supplement: Supplementary file 1 — Supplementary Information [file 41598_2017_912_MOESM1_ESM.doc]

**A novel 4-armed DNA/RNA Nanoconstruct triggering Rapid Apoptosis of Triple Negative Breast Cancer Cells within 24 hours**

Joline Tung^1†^, Lih Shin Tew^2†^, Yuan-Man Hsu^3^ and Yit Lung Khung^1*^

1. Institute of New Drug Development, China Medical University, (Taiwan)

2. Regenerative Medicine Cluster, Advanced Medical and Dental Institute (AMDI) Universiti Sains Malaysia (Malaysia)

3. Department of Biotechnology, China Medical University, (Taiwan)

**Supplementary Information**

**
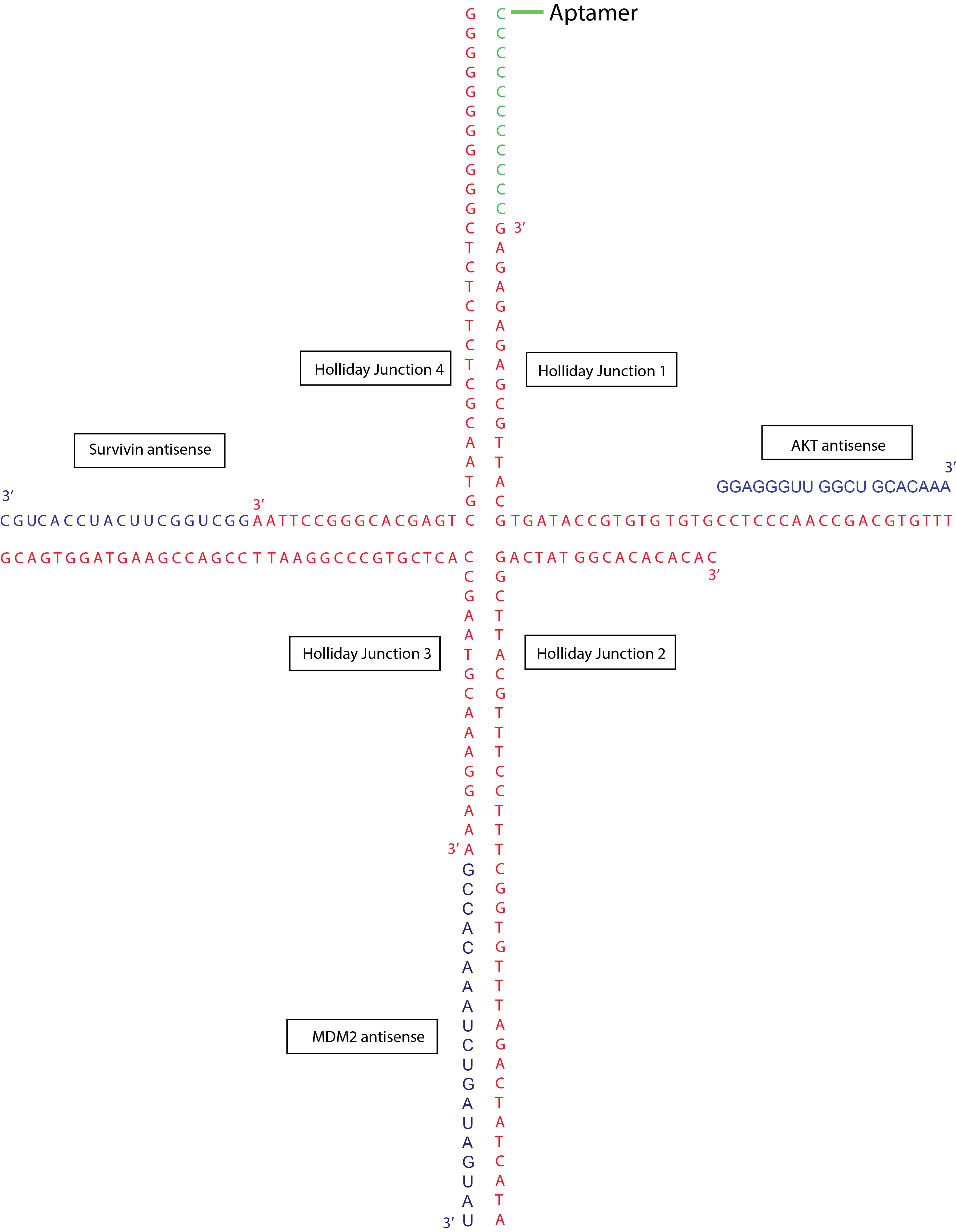
**

**Supplementary figure 1. Roadmap for the four-armed holliday junction for the simultaneous suppression of the three proteins, namely, AKT, MDM2 and survivin. The aptamer was hybridized to the nanoconstruct by means of a poly-Guanine sticky end at the top end of the nanoconstruct**

**
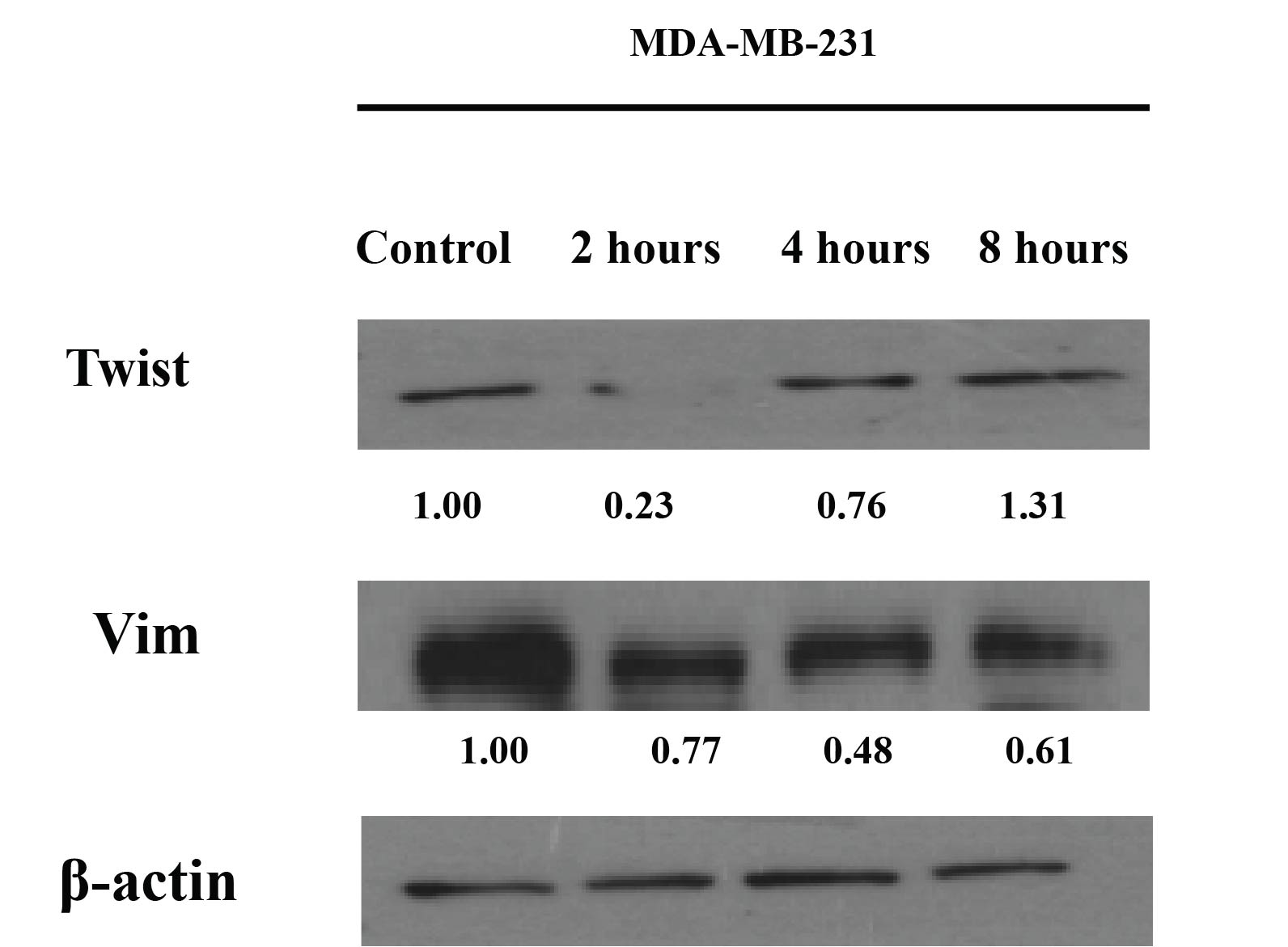
**

**Supplementary figure 2. Immunoblot analysis of cells incubated with nanoconstruct carrying three RNA antisense targeting Twist. The downstream vimentin protein was suppressed as a result of upstream Twist reduction.**

**
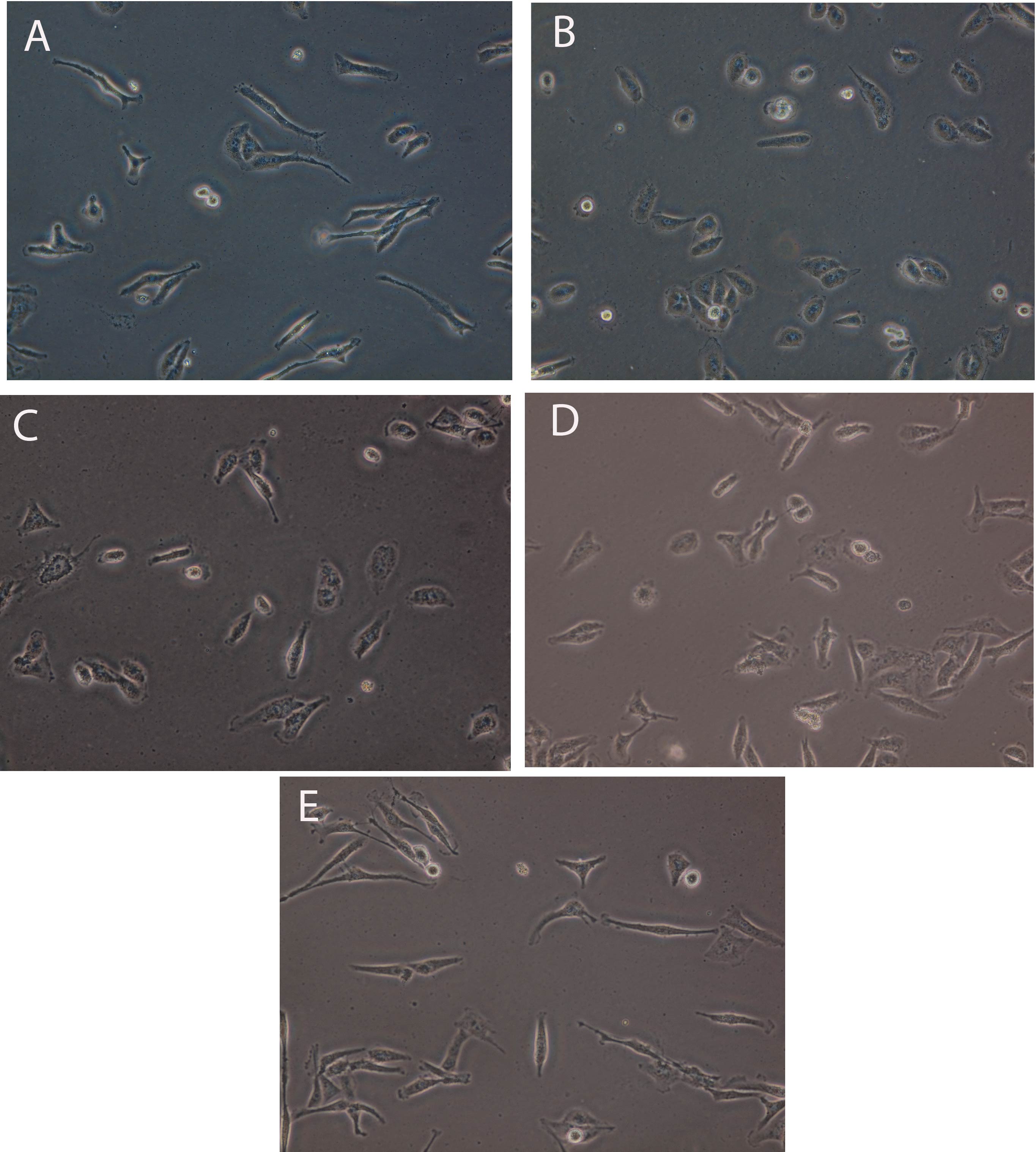
**

**Supplementary figure 3. Cell morphology after incubation with nanoconstruct carrying three Twist antisense RNA. (A) 0 hours (B) 2 hours, (C) 4 hours (D) 8 hours and (E) control at 8 hours. The uptake of the nanoconstruct had resulted in slight change cell morphology when compared to the needle-like features of normal MDA-MB-231 cells.**

**
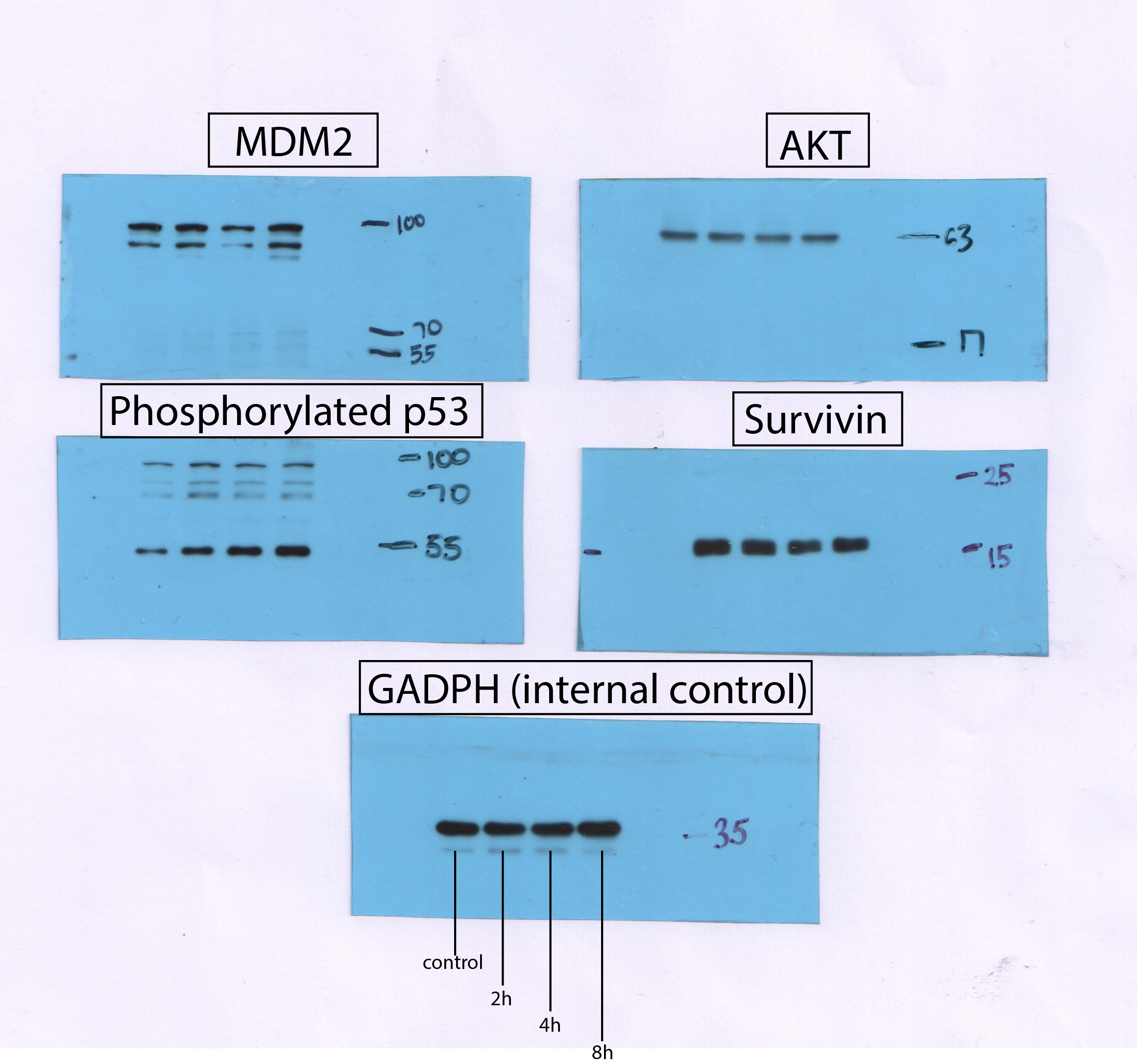
**

# Supplementary figure 4. Original images of cropped gels and blots for figure 5 in main text. All lanes within the card had been oriented from left to right, representing control, 2 hours, 4 hours and 8 hours respectively
